# Telomere length is associated with childhood trauma in patients with severe mental disorders

**DOI:** 10.1038/s41398-019-0432-7

**Published:** 2019-03-21

**Authors:** Monica Aas, Torbjørn Elvsåshagen, Lars T. Westlye, Tobias Kaufmann, Lavinia Athanasiu, Srdjan Djurovic, Ingrid Melle, Dennis van der Meer, Carmen Martin-Ruiz, Nils Eiel Steen, Ingrid Agartz, Ole A. Andreassen

**Affiliations:** 1 NORMENT K.G Jebsen Centre for Psychosis Research, Institute of Clinical Medicine, University of Oslo, and Division of Mental Health and Addiction, Oslo University Hospital, Oslo, Norway; 2 Department of Neurology, Oslo University Hospital, Oslo, Norway; 3 Department of Psychology, University of Oslo, Oslo, Norway; 4 Department of Medical Genetics, Oslo University Hospital, Oslo, Norway; 5 NORMENT, KG Jebsen Centre for Psychosis Research, Department of Clinical Science, University of Bergen, Bergen, Norway; 6 NIHR Newcastle Biomedical Research Centre & Unit, Ageing Research Laboratories, Institute of Neurosciences, Newcastle University, Campus for Ageing and Vitality, Newcastle upon Tyne, UK; 7 Department of Psychiatric Research, Diakonhjemmet Hospital, Oslo, Norway; 8 Department of Clinical Neuroscience, Centre for Psychiatric Research, Karolinska Institutet, Stockholm, Sweden

## Abstract

Reduced telomere length (TL) and structural brain abnormalities have been reported in patients with schizophrenia (SZ) and bipolar disorder (BD). Childhood traumatic events are more frequent in SZ and BD than in healthy individuals (HC), and based on recent findings in healthy individuals could represent one important factor for TL and brain aberrations in patients. The study comprised 1024 individuals (SZ [*n* = 373]; BD [*n* = 249] and HC [*n* = 402]). TL was measured by quantitative polymerase chain reaction (qPCR), and childhood trauma was assessed using the Childhood Trauma Questionnaire (CTQ). Diagnosis was obtained by the Structured Clinical Interview (SCID) for the diagnostic and statistical manual of mental disorders-IV (DSM-IV). FreeSurfer was used to obtain regional and global brain volumes from T1-weighted magnetic resonance imaging (MRI) brain scans. All analyses were adjusted for current age and sex. Patients had on average shorter TL (F = 7.87, *p* = 0.005, Cohen’s d = 0.17) and reported more childhood trauma experiences than HC (χ^2^ = 148.9, *p* < 0.001). Patients with a history of childhood sexual, physical or emotional abuse had shorter TL relative to HC and to patients without a history of childhood abuse (F = 6.93, *p* = 0.006, Cohen’s *d* = 0.16). After adjusting for childhood abuse, no difference in TL was observed between patients and HC (*p* = 0.12). There was no statistically significant difference in reported childhood abuse exposure or TL between SZ and BD. Our analyses revealed no significant associations between TL and clinical characteristics or brain morphometry. We demonstrate shorter TL in SZ and BD compared with HC and showed that TL is sensitive to childhood trauma experiences. Further studies are needed to identify the biological mechanisms of this relationship.

## Introduction

Telomeres are DNA–protein structures at the tails of chromosomes that shorten with increasing age in most human tissues.^1–3^ Human telomeres are composed of tandem repeats of the TTAGGG sequence and average between 6 and 12 kilobases (kb) in length.^1,3^ When telomeres become critically short, the risk of apoptosis is increased, and proliferation is arrested, which eventually compromises tissue renewal capacity and function.^4–6^ Telomere length (TL) may, therefore, represent a ‘molecular clock’ that contributes to aging.^2,7^ Studies report shorter age-adjusted TL in schizophrenia (SZ),^8^ bipolar disorders (BD),^9^ and in individuals at high risk for psychosis.^10^ It has been proposed that the shorter the age-adjusted TL, the more severe the illness, suggesting its potential application for clinical staging and identification of treatment needs.^11^ However, the strength of effect remains unclear as one recent study even failed to replicate the finding of shorter TL in SZ.^12^ Furthermore, the knowledge is scarce about the biological sources of potentially reduced TL in SZ/BD, and as discussed in the recent paper by Pepper et al.^13^ TL studies tend to be small and therefore underpowered.

In healthy individuals, a growing body of evidence has linked chronic stress and childhood traumatic events to accelerated shortening of TL.^14–17^ However, whether reduction of TL in SZ and in BD is linked to more frequent and more severe exposure to early trauma experiences^18^ is still not known. TL has emerged as an encouraging peripheral biomarker of cumulative stress and biological aging,^15,16^ but until now studies in SZ and BD are lacking. Patients reporting childhood traumatic events could be a subgroup of patients characterised by shorter TL, which has not yet been addressed in previous studies of SZ and BD.

On average, patients with SZ and BD have thinner cerebral cortices and smaller subcortical and cerebellar brain volumes compared with HC,^19–22^ with strongest effect sizes for the frontal and temporal lobe regions and the hippocampus. However, the mechanisms of the observed group-level brain aberrations are largely unknown. Among other factors, childhood trauma and TL may represent confounding factors, but the current knowledge is sparse regarding brain morphology and TL. A large population-based study by King et al.^23^ comprised of 1960 individuals reported a positive correlation between TL and grey and white matter volume, with the strongest associations in participants aged 50 years and older. However, King et al. did not take into account the role of having a severe mental disorder or reports of early trauma, which we aim to investigate in the current study.

Here, we assessed if patients with SZ and BD had shorter TL than HC from the same catchment area. We further investigated if childhood abuse contributed to attrition of TL in the patient group. Lastly, we tested for associations between TL and clinical characteristics, as well as key brain structures previously implicated in SZ and BD.

## Methods

### Participants

The participants were recruited from psychiatric units (outpatient and inpatient) of four major hospitals in Oslo, as part of the Thematically Organised Psychosis (TOP) Study, also called the NORMENT study. Eighty-nine percent of the sample were Caucasian. A total of 1024 participants (with SZ [*n* = 373], BD [*n* = 249]) and HC [*n* = 402]) were included. Inclusion criteria for the HC were age between 18 and 65 years and having no current or lifetime Axis 1 diagnosis from the diagnostic and statistical manual of mental disorders-IV (DSM-IV,^24^ MRI data were available from 711 individuals (SZ, *n* = 198; BD, *n* = 173 and HC, *n* = 340). Exclusion criteria for all groups included organic psychosis, neurological disorder and unstable or uncontrolled medical conditions interfering with brain function, and age outside the range of 18–65 years. The Regional Committee for Medical Research Ethics and the Norwegian Data Inspectorate approved the study. All participants gave written informed consent. The participants were enroled between 2007 and 2016.

### Clinical assessment

Trained physicians, psychiatrists and/or clinical psychologists performed clinical assessments. Diagnosis was obtained by the SCID for DSM-IV, chapters A–E. All clinical personnel completed a training programme in diagnostics and symptom rating, which was based on the training programme at UCLA (Los Angeles, California) (Ventura et al.^25^). The training programme is based on the SCID 101 training videos and includes videos with reliability testing between SCID scorers (http://www.scid4.org/index.html). The diagnostic reliability in the NORMENT sample has been found to be satisfactory with an overall agreement on DSM-IV diagnostic categories of 82% and an overall κ of 0.77 (95% CI: 0.60–0.94).

### Clinical characteristics

Number of episodes was calculated based on the SCID. Duration of illness was defined as current age minus age at the first SCID-verified episode. Current symptom level was assessed by the Positive and Negative Syndrome Scale (PANSS) (Kay et al.^26^), and current functioning was measured by the Global Assessment of Functioning scale (GAF).^27^

### Childhood trauma

To measure childhood adverse events, we used the Childhood Trauma Questionnaire (CTQ), a retrospective questionnaire that assesses traumatic experiences in childhood.^28^ The CTQ has answers ranging from ‘never true', ‘rarely true', ‘sometimes true', ‘often true', to ‘very true', and it yields a total score as well as five sub-scores: physical, emotional and sexual abuse, physical and emotional neglect^28^ (moderate-to-severe cut-off scores by Bernstein et al.^28^ were used to classify individuals has having or not having a history of childhood trauma. The reliability and validity of the CTQ have been demonstrated previously^28,29^. Owing to the high correlation between CTQ neglect items and minimisation/denial (MD) score^18,30,31^), we focused our main analyses on childhood abuse (sexual abuse, physical abuse and emotional abuse) using moderate-to-severe cut-off scores. Abuse variables (sexual abuse, physical abuse and emotional abuse) reaching moderate-to-severe cut-off score for abuse were collapsed into a total childhood abuse score.

### TLs measurement

Peripheral leucocyte TL was measured following a previously published quantitative real-time polymerase chain reaction (PCR) method with some modifications.^32^ Briefly, the abundance of telomeric template versus a single copy gene (36B4) was estimated by PCR on 10 ng of DNA, with 5 µl SYBR^®^Green JumpStart Taq Ready Mix and 0.25 µl of ROX reference dye (Sigma-Aldrich, Gillingham, UK) and the following primers: 300 nM TelA (5′-CGG TTT GTT TGG GTT TGG GTT TGG GTT TGG GTT TGG GTT-3′); 900 nM TelB (5′-GGC TTG CCT TAC CCT TAC CCT TAC CCT TAC CCT TAC CCT-3′) for the telomeric reaction and 200 nM 36B4F (5′-CAG CAA GTG GGA AGG TGT AAT CC 3′) and 400 nM 36B4R (5′-CCC ATT CTA TCA ACG GGT ACA A-3′) for 36B4. All samples were assessed in triplicate and all PCRs were carried out on an Applied Biosystems 7900HT Fast Real Time qPCR system with 384-well plate capacity. Three internal control DNA samples of known TL (10.4 kb, 3.9 kb and 2 kb) were run within each plate to correct for the plate-to-plate variation. As a further authentication of our TL measurements, we performed a revaluation of those samples that were in either the 5% top or 5% bottom of the TLs distribution as well as those samples that gave no valid data on the first run. The intra-assay coefficient of variation was 6.07% while the inter-assay coefficient of variation was 6.08%. Telomeres were defined by the ratio telomere template/amount of single-copy gene template in the direction of smaller number indicating shorter average TL. TL was measured in blood only. Blood samples were stored and extracted from the Biobank in Oslo, Norway.

### MRI data

MRI scans were obtained from three different scanners at Oslo University Hospital, Ullevål. One-hundred and seventy-eight scans were acquired using a 1.5 T Siemens MAGNETOM Sonata scanner (Siemens Medical Solutions, Erlangen, Germany) supplied with a standard head coil. For each participant, two T1-weighted images were acquired using a repeated 3D T1-weighted magnetisation prepared rapid acquisition gradient echo (MPRAGE) sequence with the following parameters: repetition time (TR) = 2730 ms, echo time (TR) = 3.93 ms, inversion time (TI) = 1000 ms, field of view (FOV) = 240 mm, flip angle (FA) = 7 o, matrix = 192 × 256, voxel size = 1.33 × 0.94 × 1 mm, 160 sagittal slices. The two T1-weighted scans obtained for each participant were averaged after rigid registration to improve signal-to-noise ratio (SNR). In total, 147 scans were obtained using a 3 T General Electric Signa HDxt scanner with an eight-channel head coil. A sagittal T1-weighted FSPGR sequence was collected with the following parameters [TR = 7800 ms; TE = 2.956 ms; TI = 450 ms, flip angle = 12 o; in-plane resolution = 1 × 1 mm; number of slices = 166; slice thickness = 1.2 mm; acquisition time = 7 min 8 s]. Lastly, 386 scans were obtained using a 3 T General Electric 750 Discovery scanner with a 32-channel head coil. A 3D IR-prepared fast spoiled gradient echo (FSPGR) T1-weighted sequence (3-D BRAVO) was collected with the following parameters: TR/TE / flip angle/voxel size/FOV/ slices/scan time = 8.16 ms, 3.18 ms, 12°, 1 × 1 × 1 mm, 256 × 256 mm, 188 sagittal slices, 4:42 min.

MRI volumes were processed using the standard FreeSurfer recon-all stream (v.5.3, http://surfer.nmr.mgh.harvard.edu), deriving anatomical segmentations of cortical and subcortical structures, and an estimate of total intracranial volume (ICV).

### Statistical analysis

Statistical analyses were performed using the IBM SPSS software, version 25.^33^ Continuous variables are summarised as mean ± SD. Analysis of covariance (ANCOVA) was applied to investigate if patients had shorter TL than controls, with and without adjusting for moderate-to-severe childhood abuse (both group status and abuse were added as fixed factors, see Table 2). Age and sex were adjusted for in the models. For the ANCOVA analyses, information on childhood abuse from the CTQ was dichotomised into < or ≥ the moderate-to-severe cut-off score by Bernstein^28^ see Supplementary Material Table S1. We computed Cohen’s d as an estimate of effect size.^34^ For the primary analyses (TL and case/control comparisons), the threshold for statistical significance was set at *p* < 0.05 with Post hoc Bonferroni corrections. Medication and TL were tested one by one, as well as a cumulative variable «TL and antipsychotic medication (yes/no) + mood stabilisers (yes/no) and antidepressant (yes/no) ».

ANCOVA was also applied to investigate the relationship between TL and clinical characteristics, as well as between TL and brain regions. For the TL and clinical analyses, the following variables were included one at a time: duration of illness, number of mood episodes, GAF and PANSS. All analyses were corrected for age, sex and diagnosis. We adjusted for number of clinical variables, yielding a significant threshold of *p* = 0.05/4 = 0.013. Brain regions were chosen based on regions previously reported to be reduced in SZ and BD.^20,22,35^ As overlapping brain region changes are reported in SZ and BD,^20,22,35^ patients were analysed as one group. As three types of scanners were used in the study, we covaried for scanner in the analyses. For the TL and brain regions analyses, we corrected for the number of brain regions investigated (10 subcortical structures, and seven regions for cortical thickness/surface area, giving a significant threshold of *p* = 0.05/17 = 0.003).

## Results

### Sample characteristics

Sociodemographic and clinical characteristics of the total sample (*n* = 1024) divided into diagnostic groups (BD, SZ) and HC are shown in Table 1. There were significantly more females in the BD group, compared with SZ or HC. Moreover, SZ were significantly younger than BD and HC. Across groups, 521 patients (84%) were taking regular current antipsychotic medication, 163 (26%) current mood stabilisers and 181 (29%) antidepressant medication. HC reported less childhood maltreatment (CTQ total score) compared with SZ and BD (X^2^ = 148.9, *p* < 0.001). Post hoc Bonferroni corrected analyses revealed no difference in reports of childhood maltreatment between the patient groups (SZ vs. BD). In the total sample, females were more likely to report at least one type of childhood abuse reaching levels of moderate- to-severe abuse by Bernstein et al.^28^ In total, 28% of all females included in the study reported childhood abuse, compared with 19% of males (X^2^ = 10.40, *p* = 0.001), see Supplementary Material Table S2.

**Table 1:** Demographics of the sample

	SZ	BD	HC	Statistics	Post hoc analyses
Age, mean ± SD	29.1 ± 9.3	31.8 ± 11.3	31.4 ± 7.6	F = 8.39, *p* < 0.001	SZ < BD, HC
Sex, *N* (%), males	221 (59)	103 (42)	228 (57)	X^2^ = 20.8, *p* < 0.001	BD < SZ, HC
CTQ, total score, mean ± SD	43.8 ± 15.5	43.3 ± 17.0	29.6 ± 5.2	F = 137.9, *p* < 0.001	HC < SZ, BD
Emotional abuse, mean ± SD	10.5 ± 0.2	10.5 ± 0.2	6.2 ± 0.2	F = 132.0, *p* < 0.001	HC < SZ, BD
Sexual abuse, mean ± SD	6.5 ± 3.3	6.7 ± 3.7	5.1 ± 0.8	F = 34.9, *p* < 0.001	HC < SZ, BD
Physical abuse, mean ± SD	7.0 ± 0.2	6.9 ± 0.2	5.2 ± 0.2	F = 45.2, *p* < 0.001	HC < SZ, BD
Daily smoking, yes (%)	220 (60)	132 (53)	−	X^2^ = 2.60, *p* = 0.11	
BMI, mean ± SD	26.6. ± 5.6	25.8 ± 4.5	−	F = 4.1, *p* = 0.04	

*CTQ* Childhood Trauma Questionnaire, *BMI* body mass index, *SD* standard deviation, n number, SZ schizophrenia, *BD* bipolar disorder, *HC* healthy controls

### TL in patients and HC

Patients (mean ± SD, 1.12 ± 0.27) had significantly shorter TL than the HC (mean ± SD 1.16 ± 0.20, F = 7.87, DF = 1, *p* = 0.005, Cohen’s d = 0.17). Post hoc tests revealed that TL was not significantly different in BD (mean ± SD, 1.13 ± 0.28) compared with SZ (mean ± SD, 1.11 ± 0.26, F = 0.68, *p* = 0.41). Within the patients, there was no association between TL and number of psychotic or mood episodes, duration of illness, GAF or PANSS (*p* > 0.1, see Supplementary Material Table S2). No significant association was observed between current medication use (antipsychotic, mood stabilisers or antidepressants) and TL (*p* > 0.1).

### TL and childhood abuse

**Fig. 1:**
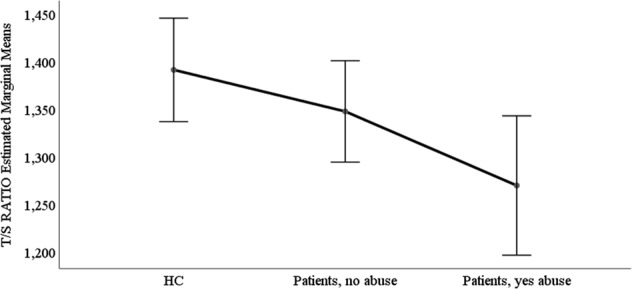
Patients with abuse have shorter telomeres than healthy controls. ANCOVA, adjusted for age and sex, F = 5.13 *p* = 0.006, partial eta squared = 0.01. Pairwise comparisons, Bonferroni adjusted; HC compared with patients with abuse: *p* = 0.003, Cohen’s *d* = 0.26; HC compared with patients without abuse: (*p* = 0.14). Patients no abuse versus patients abuse (*p* = 0.36). HC, *n* = 401; Patients without abuse, *n* = 393; and patients with abuse, *n* = 224. HC healthy controls, TLtelomere length. T/S ratio = telomere template/amount of single copy gene template. Lower score is a measure of shorter TL. Childhood trauma was defined as having at least one type of abuse reaching moderate-to-severe levels as defined by^28^

Pairwise comparisons revealed a significant difference in TL between patients with childhood abuse (mean ± SD 1.099 ± 0.26) compared with HC (F = 12.62, *p* = 0.003, Cohen’s *d* = 0.26, see Fig. 1), with no difference in TL between patients without childhood abuse (mean ± SD 1.13 ± 0.27) and HC (F = 3.73, *p* = 0.13, Cohen’s *d* = 0.11). Patients with a history of childhood abuse had shorter TL relative to HC and to patients without a history of childhood abuse (F = 6.93, *p* = 0.006, Cohen’s d = 0.16). After adjusting for childhood abuse, no statistical significant difference in TL between patients and HC was observed (F = 2.19, *p* = 0.12). Dividing into subtypes of trauma, only emotional abuse was statistical significantly associated with TL (see Supplementary Material Table 3).

### TL and subcortical regions

We found no significant relationship between TL and subcortical volumes. There was one nominally significant association with amygdala volume (F = 7.61, DF = 1, *p* = 0.01, see Table 2); however, this did not survive corrections for multiple comparisons.

**Table 2:** Telomere Length and subcortical regions

	F	DF	*p*
Amygdala	7.61	1	0.01
Hippocampus	0.92	1	0.34
Cerebellum	1.06	1	0.31
Thalamus	1.13	1	0.29
Caudate	<0.01	1	0.99
Putamen	0.05	1	0.83
Pallidum	2.52	1	0.11
Accumbens	0.09	1	0.76
Brainstem	0.97	1	0.33
Ventricles	1.23	1	0.27

Data were corrected for age, sex, ICV, group status (patients and controls) and scanner

### TL, area and thickness within regions of interest

We found no significant relationship between TL and cortical thickness or surface area after correction. Nominally significant associations were observed between TL and the superior frontal gyrus area (F = 3.73, DF = 1, *p* = 0.05), see Table 3, and frontal pole thickness (F = 2.28, DF = 1, *p* = 0.04), see Table 4.

**Table 3:** Telomere length, cortical area within regions of interest

	F	DF	*p*
Frontal pole	141	1	0.24
MFG	1.34	1	0.25
SFG	3.73	1	0.05
MTG	0.16	1	0.69
STG	0.14	1	0.71
Lat Occip	0.01	1	0.95
SPG	0.22	1	0.64

*MFG* medial frontal gyrus, *SFG* superior frontal gyrus, *MTG* middle temporal gyrus, *STG* superior temporal gyrus, *Lat Occip* lateral occipital gyrus, *SPG* superior parietal gyrus. Data were corrected for age, sex, ICV, group status (patients and controls) and scanner

**Table 4:** Telomere length and cortical thickness within regions of interest

	F	DF	*p*
Frontal pole	4.28	1	0.04
MFG	0.01	1	0.97
SFG	3.26	1	0.07
MTG	<0.01	1	0.97
STG	0.13	1	0.72
Lat Occip	0.41	1	0.52
SPG	0.02	1	0.90

*MFG* medial frontal gyrus, *SFG* superior frontal gyrus, *MTG* middle temporal gyrus, *STG* superior temporal gyrus, *Lat Occip* lateral occipital gyrus, *SPG* superior parietal gyrus. Data were corrected for age, sex, group status (patients and controls) and scanner

## Discussion

Our study confirms significantly shorter TL in a large sample of patients with SZ and BD compared with HC; however, the effect size was small. Shorter TL was specifically observed in patients reporting childhood abuse. No significant difference in TL was demonstrated between patients and HC after adjusting for childhood abuse. This suggests childhood abuse as an important factor for reduced TL in SZ and BD compared with HC.

Patients reporting childhood abuse had the shortest TL, suggesting a link between early stressors and accelerated aging,^15,36^ which until now has not been reported in SZ or BD. A history of childhood abuse has been linked to shorter TL in a smaller sample of patients with a mental illness (*n* = 290). However, this comprised of patients with a lifetime depressive, anxiety or substance use disorder, not SZ or BD.^17^ The biological mechanisms underlying the present findings are, however, unclear. Research emphasises the importance of early stressors on accelerated aging, potentially via cortisol increase of oxidative stress^14^ or cortisol reduction of blood levels of growth hormone (GH).^37^ In the past decade, the growing field of telomere science has opened exciting new avenues for understanding the cellular and molecular substrates of stress and stress-related aging processes over the lifespan. Higher attrition of TL is associated with advanced chronological age and potentially also with disease morbidity and mortality, supported by patients with SZ and BD having more than 10 years shorter life expectancy than the general population.^38,39^ An intriguing link between TL and SZ and BD could be long-term low-grade inflammation, since cell senescence increases inflammation (Freund et al.^40^); the immune system has been suggested to play a role in the pathophysiology of SZ and BD^41,42^, especially in individuals with childhood abuse.^43^ Hence, the role of TL and inflammation in SZ and BD needs further clarifications.

We found no significant associations between TL and brain structures in our study. Reduced grey matter volume reported in SZ and BD^20,22,35^ are unlikely to be related to TL, but to other underlying disease mechanisms. Our study failed to show a link between shorter TL and reduced brain regions previously shown in a population-based study.^23^ The latter study included older participants with a mean age of 50 years, compared with a median age of 29 years in our study, suggesting that TL may be more relevant for brain size in older individuals accumulating effects over time.

Our study did not show an association between clinical variables and TL. This is in contrast to Berk et al.^11^ suggesting shorter TL as a biomarker of later clinical staging, characterised by a more severe illness in BD.^11^ Similar to the study by Elvsashagen et al.^44^ we did not find an association between duration of illness and TL. Contrary to the study by Elvsashagen et al.^44^ we did not find an association between depressive episodes and TL. However, our study sample was 4–5 years younger on average and was comprised of SZ and BD while the previous study included bipolar II (BDII) only.

A few limitations to the present study should be mentioned: TL was measured in peripheral blood, and not in the brain. Data from human and non-human primates studies point to strong correlations between TL measured across somatic tissues.^45^ However, the correlation between TL in the brain and peripheral TL show inconclusive findings.^37^ The study by Dlouha et al.^46^ measuring TL in 12 different tissues, found that TL in leucocytes correlated with TL in the liver and intercostal muscle, but not TL in the brain. We also measured average TL and not number of short TL. It could be proposed that having information on the percentage of shorter TL could give more valuable information on aging and biological senescence than average TL. Moreover, childhood trauma was reported retrospectively, with the inherited weakness of the retrospective design. However, retrospective trauma questionnaires have been found to correlate with case notes,^47^ suggesting retrospective interviews as a valid instrument to collect data on childhood trauma. We also focused on abuse items and not neglect items from the CTQ since recent studies have shown that abuse items are less likely to be biased by underreporting on the CTQ questionnaire.^18,30,31^ However, it should be mentioned that reliance solely on retrospective assessment methods may have led to a proportion of non-exposed group being misclassified and thus affecting the results.^48,49^ Furthermore, due to the low percentage of HC that reported moderate-to-severe childhood abuse (*n* = 13, 3%), compared with patients (*n* = 217, 39%), we were not able to run an interaction analyses of group status and childhood trauma on TL. Since information on BMI and smoking status was limited in the control sample, we were not able to adjust for the potential effect of BMI and smoking status on TL differences between patients and healthy controls. In our study, our findings point to higher childhood abuse exposure in the patients as a mechanism for reduced TL in patients compared with HC. Brain size differences between groups tend to be small,^20,22,35^ and are thus easily influenced by several factors, including the above.

To conclude, our study demonstrates significantly shorter TL in SZ and BD and shows that TL is sensitive to stressful life events. Further studies are needed to identify the underlying biological mechanisms of this relationship.

## Supplementary information

Table S1, Table S2, Table S3
