# Supplementary material for: Telomere length is associated with childhood trauma in patients with severe mental disorders
Source: Transl Psychiatry. 2019 Mar 21;9:97. doi: 10.1038/s41398-019-0432-7 (PMC6428889; doi:10.1038/s41398-019-0432-7)
Supplement: Supplementary file 1 — Table S1, Table S2, Table S3 [file 41398_2019_432_MOESM1_ESM.docx]

**Supplementary Material**

**Table S1.** CTQ moderate to severe cutoff score for maltreatment

| CTQ, Childhood maltreatment subtypes | Moderate to severe cutoff |
| --- | --- |
| Physical abuse | ≥10 |
| Sexual abuse | ≥8 |
| Emotional abuse | ≥13 |

For estimates of frequencies of childhood maltreatment we used the moderate to severe predefined cutoff suggested by Bernstein (Bernstein and Fink, 1998).

**Table S2.** Childhood abuse divided into gender

| CTQ, Childhood maltreatment subtypes | Females | Males Statistics |
| --- | --- | --- |
| Physical abuse % | 9.7 | 9.5 χ2 =0.01 p=0.90 |
| Sexual abuse % | 17.2 | 7.5 χ2 =22.2 p˂0.001 |
| Emotional abuse % | 20.8 | 14.1 χ2 =7.8 p=0.005 |

Total sample. For estimates of frequencies of childhood maltreatment we used the moderate to severe predefined cutoff suggested by Bernstein (Bernstein and Fink, 1998).

**Table S3. Childhood trauma subtypes and TL**

|  | **TL** |
| --- | --- |
| CTQ emotional abuse | r=-0.07, p=0.03 |
| CTQ sexual abuse | r=-0.02, p=0.57 |
| CTQ physical abuse | r=-0.03, p=0.40 |
| CTQ neglect | r=-0.03, p=0.43 |

CTQ=Childhood trauma questionnaire, Spearman’s correlation; TL= telomere length. “Neglect” includes both physical and emotional neglect from the CTQ.

**Table S4.** TL and clinical features

|  | TL |  |  |
| --- | --- | --- | --- |
|  | β | T | P-value |
| Duration of illness | 0.04 | 0.80 | 0.45 |
| Number of episodes  GAF | -0.08  -0.24 | -1.60  -1.17 | 0.11  0.24 |
| PANSS Positive symptoms | -0.04 | -0.84 | 0.41 |
| PANSS Negative symptoms | -0.02 | -0.44 | 0.66 |

Analyses adjusted for sex, age, and diagnosis. GAF=Global Assessment of Functioning Scale, PANSS=Positive and Negative Syndrome Scale. n.s.= Not statistically significant. Number of episodes was adjusted for duration of illness (current age minus first SCID verified episode). TL= telomere length. Measured by telomere template/amount of single copy gene template. Lower score indicates shorter telomere length.
